# Sex-Specific Age-Related Changes in Methylation of Certain Genes

**DOI:** 10.17691/stm2021.13.3.03

**Published:** 2021-06-28

**Authors:** E.V. Kondakova, O.S. Vershinina, M.V. Lopatenko, C. Franceschi, M.V. Ivanchenko, M.V. Vedunova

**Affiliations:** Assistant, Department of General and Medical Genetics, Institute of Biology and Biomedicine; National Research Lobachevsky State University of Nizhni Novgorod, 23 Prospekt Gagarina, Nizhny Novgorod, 603950, Russia; Junior Researcher, Department of Applied Mathematics, Institute of Information Technologies, Mathematics and Mechanics; National Research Lobachevsky State University of Nizhni Novgorod, 23 Prospekt Gagarina, Nizhny Novgorod, 603950, Russia; Student, Institute of Biology and Biomedicine; National Research Lobachevsky State University of Nizhni Novgorod, 23 Prospekt Gagarina, Nizhny Novgorod, 603950, Russia; Professor Emeritus, Senior Researcher, Photonics Center, Department of Fundamental and Applied Research; National Research Lobachevsky State University of Nizhni Novgorod, 23 Prospekt Gagarina, Nizhny Novgorod, 603950, Russia; Mater Studiorum; University of Bologna, 33 Via Zamboni, Bologna, 40126, Italy; Head of the Department of Applied Mathematics, Institute of Information Technologies, Mathematics and Mechanics; National Research Lobachevsky State University of Nizhni Novgorod, 23 Prospekt Gagarina, Nizhny Novgorod, 603950, Russia; Head of the Department of General and Medical Genetics, Institute of Biology and Biomedicine; National Research Lobachevsky State University of Nizhni Novgorod, 23 Prospekt Gagarina, Nizhny Novgorod, 603950, Russia; Director of the Institute of Biology and Biomedicine; National Research Lobachevsky State University of Nizhni Novgorod, 23 Prospekt Gagarina, Nizhny Novgorod, 603950, Russia

**Keywords:** DNA methylation, CpG sites, age-associated diseases, sex-specific changes

## Abstract

**The aim of the study** was to conduct a functional analysis of sex-specific age-related changes in DNA methylation.

**Materials and Methods:**

The study used a GSE87571 methylation dataset obtained from the blood DNA of 729 individuals aged 14 to 94 using the Illumina Infinium HumanMethylation450K BeadChip (USA). Gene ontology analysis was performed for 3 groups of genes (females, males, and duplicates) using the PANTHER database. The DAVID platform was used to perform KEGG metabolic pathway analysis.

**Results:**

The studies revealed unique for males and females changes in methylation of CpG sites, associated with certain metabolic processes. It was demonstrated that most of the CpG sites, for which methylation changes with age were revealed in both sexes, are associated with the genes responsible for the development and functioning of the nervous system. In males, unique age-related methylation changes affect CpG sites associated with changes in the immune system and lipid metabolism. In females, most CpGs are associated with changes involved in transcription and translation processes. Analysis of biological functions by KEGG revealed that a unique process associated with age-related changes in methylation of the glutamatergic system is typical for males. In females, unique biological processes with age-related changes include genes responsible for the development of diabetes and genes associated with cAMP signaling cascades (KEGG:04024).

**Conclusion:**

Our studies reveal fundamental features of sex-dependent changes in methylation of CpG sites with variance increasing, which may indicate differences in age-related changes.

## Introduction

For many decades, the question of what causes the differences in life expectancy for males and females remains unresolved. Longitudinal population studies conducted in different countries and times show that females live longer than males [1]. Nowadays, the difference in life expectancy between males and females varies depending on the region of residence, reaching a maximum of 9 years in Russia. Moreover, there is an interesting paradox of the low life expectancy of males compared to females and, at the same time, a higher morbidity rate in females, which has not yet found an answer [2]. Recent reports on COVID-19 cases in different countries also indicate a sex bias in mortality rates — around 60–70% of the fatal cases were among males [3]. Sex differences remain consistent for cardiovascular disease, cancer, and neurodegenerative diseases. In particular, males have a higher incidence of myocardial infarction than females. The age-adjusted mortality from hypertension increases on average more in males than in females [4]. At the same time, some authors note that the diastolic function of the left ventricle is worse in females [5]. There is also evidence that cancer of non-reproductive tissues is more frequent in males, and mortality rates are twice as high as in females [6].

Previously proposed hypotheses regarding sex differences in life expectancy, including females’ resistance to the effects of oxidative stress, more active functioning of the immune system, the protective effect of hormones, in particular estrogen, as well as compensatory effects of the second X chromosome, have not yet been convincingly confirmed [7].

Previous studies demonstrate age-related changes in DNA methylation [8–10]. Currently, there are various methods for determining age-related changes in DNA methylation, and the relationship of these indicators with the predicted life expectancy has been shown. It is known that methylation changes are involved in the development of many age-related diseases, including oncological and neurodegenerative diseases. In this case, the search for markers of aging and the development of pathologies is of particular interest. In addition, there are many manifestations and features of a number of pathologies that are not currently characterized by sex and are not hormone-dependent. In this regard, the study of epigenetic regulation of expression in the age aspect for both sexes is of great importance. The differential assessment of hypo- and hypermethylation of various CpG sites is fundamental. Genes with an increase in variance are of particular interest since these genes are presumably associated with a slowdown or acceleration of age-dependent changes. Therefore, identification of earlier age-related changes in CpG site methylation and the study of sex differences in these processes is crucial for the early prevention of pathologies and achievement of healthy aging.

**The aim of the study** is to identify sex-specific age-related changes in DNA methylation and to conduct a functional bioinformatic analysis of these changes.

## Materials and Methods

We considered a methylation dataset GSE87571 [11] obtained using the Illumina Infinium HumanMethylation450K BeadChip (USA) on blood DNA from the Gene Expression Omnibus (GEO) datasets repository [12]. The total number of people is 729, including 341 males and 388 females aged 14 to 94 years. Probes lying on sex chromosomes, cross-reactive probes, and probes with SNPs according to list [13] were excluded from dataset. Since many early studies [14, 15] report about gender-specific in methylation, we examined the subsets of males and females separately. Gene ontology analysis was performed for 3 groups (females, males, and duplicates) genes using PANTHER database [16]. For each of the CpG lists obtained, we performed an enrichment analysis in Gene Ontology (GO) terms. Gene Ontology enrichment was performed using the methylglm function implemented in the methylGSA package [17]. Additionally, for all considered CpGs the corresponding gene symbols from the annotation for Illumina’s 450K methylation array have been converted to official gene symbols from NCBI using the alias2SymbolUsingNCBI function in the limma Bioconductor package [18].

The enrichment GO terms were categorized into MF (molecular function) and BP (biological process). More than we perform KEGG metabolic pathway analysis using DAVID platform [19]. Pathway enrichment analysis was performed on 404 and 622 unique genes from Residuals group using GSE87571. Statistics was made using Benjamini–Hochberg method taking into account FDR. The KEGG analysis results were represented as statistically significant if FDR were less than 0.05.

## Results

First of all, we identified CpG sites having significant methylation changes with age. We built for each CpG probe a linear regression model for the dependence of methylation values on age. The resulting number of CpG probes is 3827 for males and 3850 for females.

Next, we identified CpG probes with significantly age-dependent variability of methylation. Thus, we obtained 2075 probes for males and 2282 for females in which methylation and variability depend on age. For selected CpG sites, lists of corresponding unique genes (1037 for males and 1092 for females) were generated.

Additionally, blood cell counts were estimated from methylation data using Horvath calculator [20, 21]. Residuals were calculated from regression model for the dependence of beta values on counts of CD8T cells, CD4T cells, NK cells, B cells, and granulocytes. We applied the same analysis on the residuals and identified 592 probes for males and 1008 for females in which methylation and variability significantly depend on age (404 and 622 unique genes, respectively).

**Figure 1:**
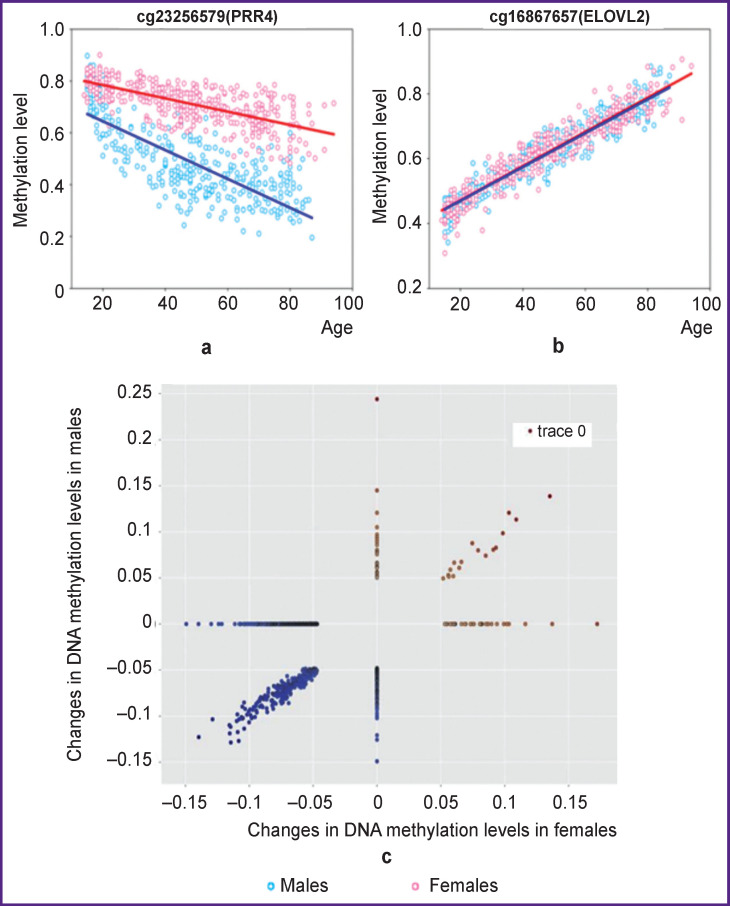
Changes in methylation levels in different sexes: (a), (b), (c) are typical examples of age-related changes in CpG site methylation

Age-dependent changes in DNA are associated with a general decrease in methylation due to a decrease in the activity of methylation enzymes. Against the background of global hypomethylation, region-specific hypermethylation is observed [22, 23]. The analysis of the selected regions showed that among the CPGs that change the level of methylation with age, there are sites that both increase methylation and decrease it (Figure 1).

We divided the lists of age-associated differentially and variably methylated positions obtained for the residuals into several categories differing in sex and direction of the residuals of methylation values (increase or decrease with age). For that, we used Scatterplot diagram of linear regression angular coefficients, age, and methylation dependence. Conventionally, 6 groups were obtained, each of which has certain features: CpGs with increasing and decreasing methylation only for females, for males, and for both sexes. The number of CpG sites in the resulting lists is presented in the Table.

**Table:** The number of CpG sites in the lists obtained depending on sex and direction (increase/decrease with age) of the residuals of methylation values

Males				Females	
592				1008	
Unique for males		Duplicates		Unique for females	
257		335		673	
Increase with age	Decrease with age	Increase with age	Decrease with age	Increase with age	Decrease with age
217	40	311	24	622	51

In the first stage of the research, we analyzed СpG sites, which demonstrated changes in methylation with age for males and females. When analyzing sex-dependent СpG specific to females, eight unique biological processes were identified (p˂0.05, FDR˂0.05). These biological processes are associated with molecular processes such as protein stabilization (GO:0050821), maintenance of protein location (GO:0045185), ribonucleoprotein complex assembly (GO:0022618; GO:0071826), spliceosomal complex assembly (GO:0000245), ubiquitin-like protein binding (GO:0032182), and also include a unique cellular component, the blood microparticle (GO:0072562).

For males, unique methylation changes are involved in 41 biological processes. They can be conventionally divided into several groups: processes associated with the maturation and differentiation of cells of the immune system (positive regulation of lymphocyte differentiation GO:0045621; alpha-beta T cell activation GO:0046631), processes associated with post-translational modification of proteins (methyltransferase activity GO:0008168; protein alkylation GO:0008213; protein methylation GO:0006479), processes of lipid biosynthesis (fatty acid derivative biosynthetic process GO:1901570; fatty acid derivative metabolic process GO:1901568; lipid transporter activity GO:0005319) and processes associated with cellular components and regulation of DNA recombination and repair (regulation of DNA recombination GO:0000018; regulation of DNA repair GO:0006282).

СpG, for which a decrease in methylation with age is shown in males, are associated with the processes of fatty acid biosynthesis and changes in ligase activity (GO:1901568 fatty acid derivative metabolic process, GO:1901570 fatty acid derivative biosynthetic process, GO:0016874 ligase activity; р˂0.05, FDR˂0.05).

CpG sites, for which an increase in methylation with age has been noted in males, are mainly related to the processes of immune system cell maturation and differentiation. Interestingly, these CpG sites are associated with the processes of post-translational protein modification (alkylation and methylation) and the activity of enzymes involved in these processes.

Analysis of sex-dependent CpG in females revealed that CpG sites showing a decrease in methylation with age are associated with the regulation of protein localization in the nucleus and the processes of protein ubiquitination. Conversely, CpG sites that increase their methylation with age in females are associated with the processes of polysaccharide biosynthesis, spliceosomal and ribonucleoprotein complex assembly, as well as the processes of regulation of proteins’ stability and maintenance of their location.

**Figure 2:**
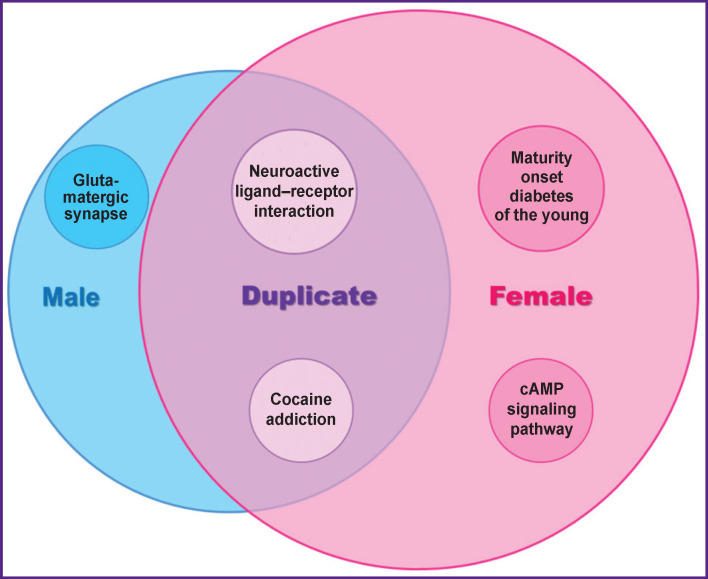
The main biological processes associated with changes in the level of CpG methylation in three groups (according to the results of KEGG analysis)

All age-dependent gender-specific genes were divided into 3 groups: genes that change methylation level with age in males (404 genes), in females (622 genes), and in both genders (“Duplicate” group) — 278 genes (Figure 2).

KEGG analysis of a duplicate genes group showed 2 most significant changes (p˂0.05) of metabolic cascades. In both sexes, age-dependent methylation of genes responsible for ligand-dependent interactions in the nervous system (neuroactive ligand-receptor interaction KEGG:04080) and genes related to the Cocaine addiction biological pathway (KEGG:05030) were found.

Analysis of the biological functions of groups of genes whose age-dependent methylation is characteristic only for females revealed five important (p˂0.05) metabolic cascades. Genes responsible for the development of diabetes (KEGG:04950) and genes associated with cAMP signaling cascades (KEGG:04024) are unique biological processes with age-dependent changes in females. In particular, there is an HTR4 gene, a member of the human family of serotonin receptors. This receptor is associated with G-protein; they stimulate cAMP production in response to serotonin. This gene’s product operates in both the peripheral and central nervous systems, modulating the release of various neurotransmitters. Its stimulation increases the production of cyclic AMP in the cell and plays a role in the regulation of memory processes, in the regulation of appetite, gastrointestinal tract functions, and mood. Recent data support the role of 5-HT4 receptors in the pathogenesis of depression, as well as in the mechanism of action of antidepressants.

The metabolic cascade of maturity onset diabetes of the young (MODY) contains genes that are crucial for the development of diabetes. In particular, the glucokinase (GCK) gene, defects in which are the cause of MODY 2. Mutations in GCK lead to chronic hyperglycemia due to decreased sensitivity of pancreatic beta cells to glucose, decreased total liver glycogen accumulation, and increased liver gluconeogenesis.

Analysis of biological functions by KEGG showed that a unique process associated with age-dependent changes in the methylation of the glutamatergic system (glutamatergic synapse (KEGG:04724)) is typical for males. It is known that glutamate is the main excitatory neurotransmitter in the central nervous system. Pathways of glutamatergic synapses, which are associated with many other pathways of neurotransmitters, play a critical role in a large number of normal physiological functions. Glutamate dysfunction is a crucial factor in many diseases of the nervous system.

## Discussion

The important feature of our research was the study of the epigenetic status of CpG sites, which change the level of methylation and variance with age and sex. Although global hypomethylation is observed with age, region-specific hypermethylation of individual DNA sites has been shown. In this study, we analyzed several subgroups of data differing in sex and direction of methylation values, including sites with both increasing and decreasing methylation values. Only CpG sites associated with intragenic regions that change both methylation and variance levels with age were included in the analysis. Differential changes in the methylation level of CpG sites, in which the variance increases in an age-dependent manner, can be considered as molecular mechanisms of differences in life expectancy between males and females [24]. Previously, a study by Yusipov et al. [25] showed a global increase in the variance of DNA methylation with aging and also indicated a tendency towards a higher variance in males compared to females in the age-related aspect. It can account for the difference in aging trajectories between the sexes.

We have shown unique for males and females changes in CpG site methylation associated with certain metabolic processes, which can determine sex-dependent age-related changes.

It was found that most of the CpG sites, for which methylation changes with age in both sexes were shown, are associated with genes responsible for the development and functioning of the nervous system. These findings are supported by literature data regarding differences between the sexes with respect to neurodegenerative diseases such as Parkinson’s disease and Alzheimer’s disease. In the first case, mortality rates in males are higher than among senior females, while the risk of Alzheimer’s disease and its mortality prevails in females [26].

We have shown that in males, age-related changes in CpG methylation are associated with genes responsible for the functioning of the immune system and lipid metabolism. Moreover, a change in lipid metabolism has much more harmful effects on males than females since even a slight increase in total cholesterol and triglycerides in males is considered a risk factor for developing atherosclerosis, while in females, such a change can be neutral due to increased metabolism of estrogen [27].

A unique biological process whose gene changes are typical for males is the functioning of the glutamatergic system. Blood glutamate levels are significantly higher in males than in females, and the system is a key regulator of drug-dependent behavior [28].

Among the unique biological processes that have age-related changes in females is the involvement of genes participating in the pathogenesis of depression. Such epigenetic changes may be the reason why age-associated depression is more common in females than in males [29].

The revealed differences in metabolic cascades associated with the development of diabetes are of interest because of the available data on the relationship between elevated glucose levels, obesity, and cancers of non-reproductive tissues. Glucose metabolism in cancer in males and females can pass through different pathways [6], thereby determining different levels of non-reproductive system cancers between the sexes.

## Conclusion

Our studies reveal fundamental features of sex-dependent changes in methylation of CpG sites with variance increasing, which may indicate differences in age-related changes.
